# Genetics of epilepsy

**DOI:** 10.3389/ebm.2026.10933

**Published:** 2026-03-19

**Authors:** Kynen Piacentini, Athanasios Gaitatzis, Sulev Kõks

**Affiliations:** 1 Perron Institute for Neurological and Translational Science, Perth, WA, Australia; 2 Centre for Molecular Medicine and Innovative Therapeutics, Murdoch University, Perth, WA, Australia; 3 Neurology Department, Sir Charles Gairdner Hospital, Nedlands, WA, Australia

**Keywords:** disease phenotype, epilepsy, epilepsy associated genes, inheritance pattern, predicted epilepsy associated genes

## Abstract

Epilepsy is one of the most common neurological diseases in the world, but it is also complex and difficult to study. There is a significant genetic component to epilepsy and more information is being published frequently. It is difficult to group and summarise all of this information in a way that is beneficial for both researchers and clinicians. The aim of this paper is to create a summary of all currently known epilepsy associated genes in order to aid epilepsy research to better understand the aetiology of the disease. This was achieved through gathering genetic data from three databases: Online Mendelian Inheritance in Man (OMIM), Clincal Genome (ClinGen), and PubMed. Genes were filtered based on specific criteria and were summarised into three tables: Epilepsy genes, Epilepsy associated genes and Predicted epilepsy associated genes. A fourth table was produced to showcase all epilepsy genes that were identified in all three databases. A total of 2,536 genes were identified to have some level of association with epilepsy. A total of 238 genes were classified as Epilepsy genes, 1,317 genes were classified as Epilepsy associated genes and 981 genes were classified as Predicted epilepsy genes. Finally, 86 genes were identified to be epilepsy genes that were found in all three genetic databases and represent the highest confidence in association with epilepsy. The significance of this study involves the ability to give researchers an up-to-date list of genes that have an association to epilepsy and a summary of information about said genes.

## Impact statement

Epilepsy is a complex neurological disease that involves a huge genetic component that is not fully understood. This study provides a current list of all genes that have some association with epilepsy by grouping each gene on their specific association level. This study identified a total of 2,536 genes that have some level of association with epilepsy compared to a study by Zheng MW, et al. [1] who identified a total of 1506 genes to be associated with epilepsy back in 2023 (published in 2024). This study provides mode/s of inheritance details for each gene, phenotypic details of each gene (Supplementary Table S1) and an overall classification. With this information, researchers are able to gain an insight on an up-to-date catalogue of epilepsy associated genes in which to base their future studies on. Recognition of the genes associated with epilepsy will benefit future screening strategies and aid in the discovery of the precise clinical molecular diagnosis and how we manage epilepsy.

## Introduction

Neurological diseases are inherently complex due to the intricate anatomy and function of the nervous system, the presence of multiple causal and contributing factors, diverse symptoms and diagnostic challenges. Many of these diseases are heritable, meaning that genetic factors play a large role in their development, expression and progression. Epilepsy is the second most prevalent chronic neurological disease with thousands of associated genes contributing to its diverse clinical manifestations, with new genetic links to different types of epilepsy uncovered every year [2]. Advances in next-generation sequencing technologies and bioinformatics have accelerated the discovery of new epilepsy associated genes and our understanding of the disease. Many papers have previously analysed the total number of genes that are associated with epilepsy. In 2017, Wang et al. [3] identified 977 genes that were associated with epilepsy based on clinical-genetic evidence. In 2024, Zhang MW, et al. [1] almost doubled this number identifying 1506 genes that were associated with epilepsy based on the same clinical-genetic evidence. Over the past several years a significant number of epilepsy associated genes has been identified. Many novel genes have been introduced and many are no longer considered to be associated with epilepsy. With an ever changing and evolving list of epilepsy associated genes, studies need to be continuously updated to aid epilepsy research. Many previous papers have focussed on providing a list of genes that can be potentially useful in clinical genetic diagnosis and precise treatment of epilepsy. To update the current list of epilepsy associated genes we utilised three major databases, this includes the Online Mendelian Inheritance in Man (OMIM) database, PubMed and ClinGen. The purpose of this study is to group together all genes potentially associated with epilepsy to specifically aid genetics research in the field of epilepsy.

## Methods

To update the list of genes associated with epilepsy, a systematic study was conducted based on data from several online databases until November 2025. After obtaining and filtering through the list of genes identified from the OMIM, ClinGen and PubMed database, genes were grouped into three categories: “Epilepsy genes” (Table 1), “Epilepsy associated genes” (Table 2) or “Predicted epilepsy associated genes” (Table 3) based on independent criteria for each database. Requirements for each category can be found in Supplementary Table S1. A table was produced for each category showcasing the current list of genes associated with epilepsy as well as the mode of inheritance and phenotype. Another table (Table 4) was created to summarise all epilepsy genes that were identified in all databases that includes each genes phenotype/s and mode/s of inheritance. The phenotypes found in Table 4 were database derived and formatted and confirmed manually.

Genes were first searched in the OMIM database^1^ using the following terms: epilepsy, epilepsies, epileptic, convulsion, convulsions, seizure, seizures. The OMIM database utilises a phenotype map key to further characterise each gene. We took advantage of this key to categorise genes into their respective categories. The phenotype map key is comprised of four types, this includes [2] there is a gene-disease relationship, however the underlying genetic defect is unknown according to the University of California Santa Cruz (UCSC) genome browser [3]; the gene has been included through linkage analysis (mapping studies), however a specific gene mutation has yet to be identified [1]; the molecular basis of the disorder is known and a mutation has been identified [4]; the disorder is a contiguous gene deletion or duplication syndrome (multiple genes are deleted or duplicated) according to the UCSC genome browser. Genes from the OMIM database were categorised as “Epilepsy genes” if they had a phenotype map key of 1 and the phenotype included at least one of the key words that was originally searched. Genes were categorised as “Epilepsy associated genes” if the phenotype map key was 1,3 and/or 4 and the phenotype did not include one of the epilepsy key words. Finally, genes were grouped into the “Predicted epilepsy associated genes” category if the phenotype map key was 2 or blank and the phenotype did not include one of the epilepsy key words.

ClinGen^2^ was the second database utilised where we included all genes in the gene-disease validity section. Genes were grouped into the three categories based on their classification and phenotype. Classifications included disputed, limited, moderate and definitive. Genes were immediately excluded if their classification was disputed. Only genes with one of the epilepsy key words as previously mentioned were included. Genes were added to the current “Epilepsy genes” list if they were classified as definitive, genes were added to “Epilepsy associated genes” if they were classified as moderate and genes were added to “Predicted epilepsy associated genes if they were classified as limited.

The final database utilised was PubMed^3^ where we followed a similar procedure as the OMIM database. Genes were added to the list based on if the key words were included in its respective phenotype. Only genes found in *homo sapiens* were recorded for this study. Genes that did not included one or more of the epilepsy key words were assigned to the Epilepsy associated genes group. We understand that the PubMed database is a literature database rather than a curated gene-disease resource. To resolve this, genes found only within the PubMed database, the highest level of classification they received was “Epilepsy associated genes.” Priority was given to the other databases OMIM and ClinGen as they are curated gene-disease resources. To resolve conflicting classifications across the three databases, a predefined hierarchical evidence framework was applied. Gene categories were ranking according to confidence level where the highest confidence classification was “Epilepsy genes” and the lowest was “Predicted epilepsy associated genes”. When a gene was assigned to multiple categories across different databases, the highest confidence classification was retained as the final group. This approach ensured consistent prioritisation based on evidence strength rather than source frequency.

## Results

**FIGURE 1:**
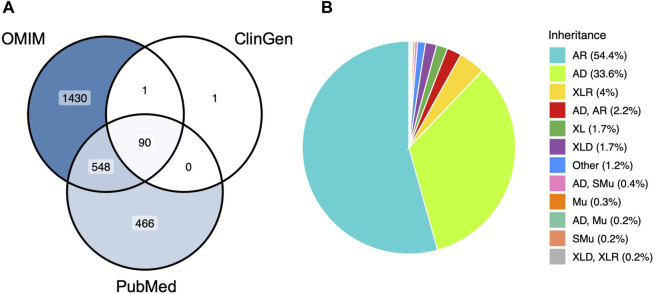
The overall organisation of epilepsy associated genes. **(A)** Distribution of epilepsy associated genes between databases after filtering. **(B)** Distribution of the 11 most common modes of inheritance from the 2536 epilepsy associated genes. AR: autosomal recessive; AD: autosomal dominant; XL: X-linked; XLR: X-linked recessive; XLD: X-linked dominant; SMu: somatic mutation; Mu: multifactorial.

The present study filtered through a list of 3686 epilepsy-associated genes from OMIM, 1977 epilepsy-associated genes from PubMed and 3334 genes from ClinGen and grouped the genes according to their potential associated with epilepsy and the mode of inheritance. These genes included 238 epilepsy genes, 1317 epilepsy associated genes, and 981 predicted epilepsy associated genes, which were recruited from OMIM, and genes were added from ClinGen and PubMed accordingly. The distribution of these genes between the databases is illustrated in Figure 1A and the distribution of the modes of inheritance is illustrated in Figure 1B. Therefore, based on the criteria of this study, a total of 2536 genes currently have some association with epilepsy.

### Epilepsy genes

Overall, 238 genes were classified as epilepsy genes compared to that of 168 epilepsy genes found by Zhang MW, et al. [1]. These 238 genes were listed according to their mode of inheritance as stated within each genetic database, many of which have multiple modes of inheritance. Most epilepsy genes (58.0%) were of autosomal recessive inheritance; 48.7% genes were of autosomal dominant inheritance; and 4.2% genes were of X-linked inheritance. Notable phenotypes for epilepsy genes include encephalopathy (43.7%), neurodevelopmental disorder (23.5%), microcephaly (8.4%), myoclonic (8.0%), and hypotonia (7.1%). All genes found in this category suggested a mode of inheritance that was either autosomal or X-linked, except for PIGT. This gene included the mode of inheritance, somatic mutation.

**TABLE 1:** Epilepsy genes.

Inheritance	Genes
AD	*AGO1, AP2M1, ARFGEF1, ATN1, ATP1A1, ATP1A3, ATP6V0C, CACNA1A, CACNA1C, CACNA1E, CACNA1I, CDK19, CELF2, CHD2, CHRNA2, CHRNA4, CHRNB2, CUL3, CUX2, CYFIP2, DHX16, DLL1, EEF1A2, FBXO28, FGF12, FZR1, GABRA1, GABRA5, GABRB1, GABRB2, GABRB3, GABRG2, GNAO1, GRIA4, GRIN2A, GRIN2B, GRIN2D, HCN1, HECW2, HNRNPU, IRF2BPL, KCNA2, KCNB1, KCNC1, KCNC2, KCNH5, KCNK4, KCNQ2, KCNT1, KCNT2, LGI1,* ***MARCHF6*** *, MAST3, NACC1, NBEA, NCDN, NEUROD2, NPRL2, NPRL3, NSF, NTRK2, PACS2, PAK1, PHACTR1, PHF21A, PPP3CA, PRRT2, RHOBTB2, RNF13, RORA, SAMD12, SCN1A, SCN2A, SCN3A, SEMA6B, SERPINI1, SETD1A, SETD1B, SIK1, SLC1A2, SLC32A1, SLC6A1, SNAP25, SPTAN1, STARD7, STX1B, TANC2,* ***TCP1*** *, WASF1, YWHAG*
AD | AR	*ATP6V0A1, CACNA1D, CLCN3, EXT2,* ***EIF4A2*** *, GLS, KCNQ3, TBC1D24, ATP1A2, GLUL, KCNMA1, DIAPH1, DNM1, SCN1B, STXBP1, CPA6, ACTL6B, ATP6V1A, DEAF1, GRIK2, GRIN1, DEPDC5, NARS1, NUS1*
AD | AR | SMu	*PIGT*
AR	*ABCA2, ADAM22, ADARB1, ADPRS,* ***AFG2A*** *, ALDH7A1, AP3B2, ARV1, ASAH1, BRAT1, CACNA1B, CACNA2D1, CACNA2D2, CAD, CERS1, CHKA, CLN8, CNPY3, CNTN2, CPLX1, CPSF3, CSTB, DENND5A, DHPS, DOCK7, EMC10, EPM2A, ESAM, EXOC7, FRRS1L, GAD1, GOSR2, GOT2, GRM7, GTF3C3, HACE1, HECTD4, HID1, IER3IP1, ITPA, KCTD7, LIAS, LNPK, MDH2, MED11, MED17, MED23, MTHFS, NAPB, NECAP1, NHLRC1, NRROS, NSRP1, OTUD6B, OTUD7A, OXR1, P4HTM, PARS2, PDE2A, PI4K2A, PIGB, PIGF, PIGK, PIGN, PIGP, PIGQ, PIGS, PIGU, PLCB1, PLPBP, PNKP, PNPO, PPFIBP1, PRICKLE1, PRMT7, PTPN23, QARS1, RTN4IP1, RTTN, SARS1, SCARB2, SLC13A5, SLC25A12, SLC25A22, SLC31A1, SLC35A3, SLC38A3, SLC7A6OS,* ***SNF8*** *, SNIP1,* ***SPOUT1*** *, ST3GAL3, STRADA, SV2A, SYNJ1, SZT2, TBC1D2B, TIAM1, TRAK1, TRAPPC4, TRAPPC6B, TUBGCP2, UBA5, UGDH, UGP2, VARS1, VPS50, WARS2, WDR4, WDR45B, WWOX, YIPF5*
XL	*ALG13, ARHGEF9, GABRA3, PCDH19, SYN1*
XLD	*CDKL5, SMC1A*
XLR	*PIGA, ARX*
XLD | XLR	*FGF13*

AD, autosomal dominant; AR, autosomal recessive; XL, X-linked; XLD, X-linked dominant; XLR, X-linked recessive; DR, digenic recessive; Mu, multifactorial inheritance; SMu, somatic mutation; SMo, somatic mosaicism; DD, digenic dominant; PR, psuedoautosomal recessive; UN, unknown. Genes that have been identified in this study that were not present within the Zhang MW, et al. [1] have been highlighted in bold.

### Epilepsy associated genes

A total of 1317 genes were categorised as epilepsy associated genes in this study compared with the study by Zhang MW, et al. [1] who identified 974 epilepsy-related genes. These 1317 genes were listed according to their mode of inheritance as stated within each genetic database with many having multiple modes of inheritance. Most epilepsy genes were of autosomal recessive inheritance (65.1%); 34.1% genes were of autosomal dominant inheritance; and 8.2% genes were of X-linked inheritance. Notable phenotypes for epilepsy genes include mitochondrial dysfunction (8.9%), neurodevelopmental disorder (6.8%), microcephaly (3.6%), encephalopathy (2.5%) and hypotonia (2.5%). The gene DHRSX was the only gene in the entire study to have a suggested mode of psudoautosomal recessive.

**TABLE 2:** Epilepsy associated genes.

Inheritance	Genes
AD	*ACTA2, ACTB, ACTG1, ACVR1,* ***ACVRL1*** *, ADGRL1, ADNP, AFF3, AGO2, AHDC1, AKT2, AKT3, ANK2, ANKH, ANKRD11, ANKRD17, APP, ARCN1, ARF1,* ***ARHGAP31*** *, ARID1A, ARID1B, ARID2, ASH1L, ASXL1, ASXL2, ASXL3, ATP2B1, ATP6V1B2, ATXN10, AUTS2, BCL11B, BICD2, BICRA, BMP4, BRAF, BRPF1, CACNA1G,* ***CALM1*** *, CALM2, CAMK2B, CAMK2G, CAMTA1,* ***CAPRIN1*** *, CCM2,* ***CCT3*** *,* ***CD96*** *, CDC42, CDC42BPB, CDK13, CDK8, CEP85L, CERT1, CHAMP1,* ***CHASERR*** *, CHD1, CHD3, CHD5, CHD8, CHN1, CIC, CLCN6, CLDN11, CLTC, CNOT1, CNOT3, CREBBP, CSNK2A1, CSNK2B, CTNNB1, CUX1,* ***CYP2C9*** *, DAGLA,* ***DDX6*** *, DHX30,* ***DHX9*** *, DIP2B, DLG4,* ***DMPK*** *, DNAJC5, DNMT1, DNMT3A, DPYSL5,* ***DUSP6*** *, DYNC1H1, DYRK1A, EBF3, EED, EFTUD2, EHMT1, ELN, ENG, EZH2, FAM111A, FBXO11,* ***FBXW11*** *, FBXW7,* ***FGF14*** *, FGF8, FGFR1,* ***FOSL2*** *, FOXG1, FOXP1,* ***FOXP2*** *, FRMD5,* ***FRYL*** *, FXYD2, GABBR1, GARS1, GATA3, GATA6, GATAD2B, GFAP, GLI2, GLI3, GLUD1, GNA11, GNAI1,* ***GNAS*** *, GNB1,* ***GPRC5B*** *, GRIA2, H3-3A, H3-3B, H4C3, H4C5, HDAC4, HIVEP2,* ***HNRNPC*** *,* ***HNRNPH1*** *,* ***HNRNPR*** *,* ***HRAS*** *,* ***JPH3*** *, KANSL1, KAT5, KAT6A, KAT8, KCNA1, KCND3, KCNE2, KCNH1, KCNH2, KCNJ2, KCNJ5, KCNJ6, KCNQ5, KDM3B, KDM4B, KDM6B, KIF11, KIF2A, KIF5C, KMT2A, KMT2C, KMT2D, KMT2E, KMT5B, KRAS, KRIT1, LMBRD2, LMNB1, LMX1B, LRP12,* ***LRRC8C*** *, MACF1, MAF, MAGEL2, MAP2K1, MAPK1, MAPK8IP3, MAPRE2, MAST1, MATR3, MBD5, MED12L, MED13, MED13L, MEF2C, MEN1,* ***MIB1*** *, MN1,* ***MSL2*** *, MSX2, MTOR, MTSS2,* ***MYCN*** *, MYOCD, MYRF, MYT1L, NAA15, NEDD4L, NFE2L2, NFIA, NFIB, NIPA1, NIPBL,* ***NLRP3*** *, NOTCH1, NOTCH2, NOTCH2NLC, NOVA2, NPTX1, NR2F1, NR4A2, NSD1, NSD2, OTX2, PACS1, PAFAH1B1,* ***PANX1*** *, PAX2,* ***PDCD10*** *,* ***PDGFB*** *, PDYN, PIK3R2,* ***PNKD*** *, POGZ, POLR2A, POU3F3, PPFIA3, PPP1R12A, PPP2CA, PPP2R1A,* ***PPP2R5C*** *, PPP2R5D, PRKAG2, PRKAR1B, PROK2, PROKR2,* ***PRPF6*** *,* ***PRR12*** *, PSEN2, PSMD12, PTCH1, PTPN11, PUF60, PUM1, PURA, RAB11B, RAC1, RAC3, RALA,* ***RAP1B*** *, RFX7, RNF125, RNF2,* ***RNU4-2*** *, RRAGD, RYR2, SATB1, SATB2, SCAF4,* ***SCN2B*** *, SET, SETBP1, SETD2, SETD5,* ***SF3B2*** *, SGCE, SIAH1, SIN3A, SIX3,* ***SLC17A8*** *, SLC1A3, SLC20A2, SMAD2,* ***SMAD4*** *, SMARCA2, SMARCC2,* ***SMARCD1*** *,* ***SNRPE*** *, SNTA1, SON, SOX2, SOX4, SOX5,* ***SOX6*** *, SPAST, SPEN, SPOP, SPTBN1, SPTLC2, SRCAP, SRP54,* ***SRSF1*** *, STAG1,* ***STOX1*** *, SUPT16H, SYNGAP1,* ***SYT1*** *,* ***TAF4*** *, TBL1XR1, TBX1, TCF20, TCF4,* ***TEK*** *, TLK2, TMEM106B,* ***TMEM151A*** *, TMEM163, TMEM63A, TNPO2, TOP2B, TRAF3, TRAF7, TRIM71, TRIM8, TRIO, TRIP12, TSC1, TUBA1A,* ***TUBB*** *, TUBB2A, TUBB2B, TUBB3, TUBB4A, TUBG1, U2AF2,* ***UBAP2L*** *, UBE3A, UBTF, USP7, VAMP2, VPS4A, WAC, WDR26, WDR37, XPR1, ZBTB18, ZEB2, ZMIZ1, ZMYM2, ZMYND11, ZNF292, ZSWIM6*
AD | AR	*ABCC8, FGFR3, FTL, GCM2, KCNJ11, SLC16A1, CRYAB, ACOX1, AFG3L2, ALDH18A1, AP1G1,* ***CFAP43*** *, CLCN1, CLPB, COL18A1, COL3A1, DCHS1, DHX37, DSTYK, ERLIN2, FAR1, GCK, GRM1, HPD, HTT, IFIH1, KIF1A, MFN2,* ***MYH3*** *, NALCN,* ***PLG*** *, POLR1A, POLR3B, PPOX, PROC, PROS1, RBP4,* ***SAMD9*** *, SCN9A,* ***SCNN1G*** *, SLC12A2, SLC25A4, SQSTM1, STAT1, STT3A, STUB1, THRB, YARS1,* ***AQP2*** *,* ***DISP1*** *, GLRA1, HESX1, OPLAH, POLRMT, PTH,* ***SPG7*** *, TET3, ZFP57, DNM1L, EIF2AK2, ITPR1, ALPL, ATAD3A, GCH1, GRN, ALG8, AP4E1, BSCL2, COG4, CSF1R, ELOVL4,* ***EVC2*** *, FH,* ***GH1*** *, GJA1,* ***GNE*** *, HEPACAM,* ***HK1*** *, HMBS, HSPD1, INSR, KCNE1,* ***KLHL24*** *, KLHL7, OPA3, ORAI1, PARN, PDE10A, PNPT1, POLG, POMP, PPP2R3C,* ***RHAG*** *, RRM2B, RTN2, RYR3, SCN4A, SCO2, SDHA, SDHD, SLC12A6, SLC33A1, TWNK, WARS1,* ***CHRNA1*** *, ACO2, PEX6*
AD | AR | DD	*ADGRV1*
AD | AR | IC	*SDHB*
AD | DD	*CAV3*
AD | IC	*RAI1*
AD | SMo	*PRKACB*
AD | SMu	*NF1*
AR	*AASS, ABAT, ABHD12, ABHD16A,* ***ACACA*** *,* ***ACAD8*** *, ACADM, ACADS, ACADSB,* ***ACBD6*** *, ACSF3, ACY1, ADA2, ADAMTSL2,* ***ADAT3*** *, ADD3, ADGRG1, ADK, ADSL,* ***AFG2B*** *, AGA, AGL, AGPS,* ***AHCY*** *, AHI1, AIMP1, AIMP2, ALDH3A2, ALDH4A1, ALDH5A1, ALDOB, ALG1, ALG11, ALG12, ALG2, ALG3, ALG6, ALG9, ALKBH8, AMACR, AMFR, AMT, ANK3, ANKLE2, ANO10, AP4B1, AP4M1, AP4S1, APC2, ARFGEF2, ARG1, ARHGDIA, ARL6IP1, ARMC9, ARSA, ASL, ASNS, ASPA, ASPM, ASS1, ATAD1, ATG7, ATIC, ATP13A2, ATP5F1D, ATP5F1E, ATP5PO, ATP6V0A2, ATP9A, AUH, B3GALNT2,* ***B3GAT3*** *,* ***B4GALT1*** *, B4GAT1, BCAS3, BCKDHA,* ***BCKDHB*** *, BCKDK, BCS1L,* ***BET1*** *,* ***BIN1*** *, BLOC1S6,* ***BLTP1*** *, BOLA3,* ***BORCS8*** *, BTD, C12orf57, C2orf69,* ***CA8*** *, CAMSAP1, CAPN15,* ***CAPNS1*** *, CARD9,* ***CARS1*** *, CARS2,* ***CASP2*** *, CASQ2,* ***CBS*** *, CC2D1A, CC2D2A, CCBE1, CCDC88A, CD59, CDK10,* ***CDK5RAP2*** *,* ***CEP135*** *,* ***CEP152*** *, CEP164, CHKB, CIT, CKAP2L, CLDN16, CLN3, CLN5, CLN6, CLP1, CLPP, CNTNAP1,* ***COA8*** *, COASY,* ***COG3*** *, COG5, COG6, COG7, COLGALT1, COPB1, COQ4, COQ6, COQ8A, COQ9, CORO1A, COX10, COX11, COX15, COX4I1, COX6B1, CPT1A, CRADD, CRB2, CRBN, CRIPT, CRLF1, CRLS1, CSPP1, CTC1, CTH, CTNNA2, CTSA, CTSD, CTSF,* ***CTU2*** *, CYB5R3, CYP27A1, CYP27B1, D2HGDH, DAP3, DARS2, DBH, DBT, DCAF17, DDHD2, DDX59, DEGS1,* ***DGUOK*** *, DHCR24,* ***DHCR7*** *, DHFR,* ***DIS3L2*** *, DLD, DNAJC12, DNAJC21, DNAJC6,* ***DOCK3*** *, DOCK6, DOCK8, DOHH, DOLK, DPAGT1, DPH1, DPH5, DPM1, DPM2, DPP9, DPYD, DPYS,* ***DRG1*** *,* ***DST*** *, DTYMK, DYNC1I2, EARS2, ECHS1, ECM1,* ***EDEM3*** *,* ***EEF1D*** *,* ***EEFSEC*** *, EIF2B1, EIF2B2, EIF2B3, EIF2B4, EIF2B5, EIF3F, ELP2, EMC1,* ***EMG1*** *, EML1, ENTPD1, EPG5,* ***EPRS1*** *,* ***ERCC8*** *, ESCO2,* ***ETFA*** *,* ***ETFB*** *, ETFDH, ETHE1, EXOC2, EXOSC3, EXOSC5, EXOSC9, EXTL3, FA2H, FADD,* ***FAM177A1*** *, FANCA, FARS2, FARSB, FASTKD2, FAT4, FBP1, FBXL4, FBXO22, FCHO1, FCSK, FDFT1,* ***FDXR*** *,* ***FILIP1*** *,* ***FITM2*** *, FKTN,* ***FLVCR1*** *, FLVCR2, FMN2, FOLR1, FOXRED1, FRA10AC1,* ***FUCA1*** *, FUT8,* ***FXN*** *, GALC, GALNT2, GAMT, GCDH, GCSH, GEMIN4, GFER, GFM1, GFM2,* ***GIPC3*** *, GLB1, GLDC, GLE1, GLRB,* ***GLRX5*** *, GLYCTK, GM2A, GMPPB, GNB5, GNPAT,* ***GNS*** *, GOLGA2, GPAA1, GPHN, GPSM2, GPT2, GPX4,* ***GRID2*** *, GSS,* ***GSX2*** *,* ***GTPBP1*** *, GTPBP2, GTPBP3,* ***GUCY1A1*** *, GYS1, GYS2,* ***HADH*** *, HADHA, HADHB, HAX1, HERC1, HEXB, HGSNAT, HHAT, HIBCH, HINT1, HLCS, HMGCL, HMGCS2,* ***HOXA1*** *, HPDL, HSD17B4, HYCC1, IARS1, IARS2, IDH3A, IFNAR1,* ***IL7*** *,* ***IMPA1*** *, INPP5K, INTS1, INTS11, IQSEC1, IREB2, ISCA1, ISCA2, ISG15,* ***IVD*** *, JAM2, JAM3, KATNB1,* ***KCNJ1*** *, KCNJ10,* ***KCNJ16*** *, KDM5A, KDM5B,* ***KICS2*** *,* ***KIF26A*** *, KLC2, KNL1, KPNA7, KPTN, L2HGDH, LAMA2, LAMB1, LAMC3, LARS2, LCP2,* ***LDHD*** *, LETM1, LGI3, LGI4, LIG3, LINGO1, LINS1, LIPT2, LMBRD1, LONP1, LRP2, LRPPRC,* ***LRRK1*** *, LSS,* ***LYST*** *, MAD1L1, MADD, MAGI2, MAN1B1,* ***MAN2B2*** *, MANBA, MAPKAPK5, MBOAT7,* ***MC2R*** *, MCCC1, MCCC2, MCM3AP,* ***MCPH1*** *,* ***MECR*** *,* ***MED16*** *, MED25, MED27, MEGF10, METTL23, METTL5, MFF, MFSD2A, MFSD8, MGAT2, MGP, MICOS13, MICU1, MIPEP, MKS1, MLC1, MLYCD, MMAA, MMACHC, MMADHC, MOCS1, MOCS2, MOGS, MPC1, MPDU1, MPDZ, MPI, MPV17, MRAP, MRM2,* ***MRPL39*** *,* ***MRPL49*** *, MRPS22, MTFMT, MTO1, MTX2, MYMK, MYO5A, MYORG,* ***NAA20*** *,* ***NAA60*** *, NADK2, NAE1, NAGA,* ***NAGS*** *, NANS,* ***NAV3*** *, NAXD, NAXE, NBAS, NCAPD3, NDE1, NDST1,* ***NDUFA11*** *, NDUFA2, NDUFA6, NDUFA8, NDUFAF2, NDUFAF3, NDUFAF4, NDUFAF5, NDUFAF6, NDUFAF8, NDUFB8, NDUFC2,* ***NDUFS1*** *, NDUFS3, NDUFS4,* ***NDUFS6*** *, NDUFS7, NDUFS8, NDUFV1, NDUFV2, NEU1,* ***NEUROG1*** *, NFASC, NFS1, NFU1, NGLY1,* ***NHLRC2*** *, NNT, NPC1, NPC2, NRCAM, NSUN2, NSUN3,* ***NSUN6*** *,* ***NT5E*** *, NTNG2, NTRK1, NUBPL,* ***NUP188*** *,* ***NUP62*** *, OCLN, OGDH, OSGEP, OSTM1, PANK2, PC, PCCA, PCCB, PCDH12, PCDHGC4, PCK1, PCLO, PCNT, PCYT2, PDHB, PDHX, PDP1,* ***PDSS1*** *, PDSS2,* ***PDXK*** *, PDZD8, PET100, PEX1, PEX10, PEX12, PEX13, PEX14,* ***PEX16*** *, PEX19, PEX2, PEX26, PEX5, PEX7,* ***PFKM*** *, PGAP1, PGAP2, PGAP3, PGM1, PGM2L1, PGM3, PHGDH, PHKG2, PI4KA, PIBF1, PIDD1, PIGC, PIGH, PIGL, PIGM, PIGO, PIGV, PIGW, PIGY,* ***PINK1*** *,* ***PISD*** *, PITRM1, PKHD1L1, PLA2G6, PLAA, PLCH1, PLEKHG2,* ***PLK4*** *,* ***PLOD3*** *, PLVAP, PLXNA1, PMM2, PMPCB, POLR3A, POLR3K, POMGNT1, POMT1, POMT2, PPCS, PPIL1, PPP1R15B,* ***PPP1R21*** *, PPT1, PRDM13, PRDX1, PRF1, PRKDC, PRKN,* ***PROP1*** *, PRORP, PRUNE1, PSAT1, PSMB8, PSPH,* ***PTCD3*** *,* ***PTF1A*** *,* ***PTPMT1*** *, PTRH2,* ***PTRHD1*** *, PTS,* ***PUS1*** *, PUS3, PYCR2, QDPR, RAB18, RAB27A, RAB3GAP1, RAB3GAP2, RALGAPA1,* ***RAP1GDS1*** *, RAPSN, RARS1, RARS2, RBCK1, RBL2, RBM8A, RBSN,* ***RECQL4*** *, RFT1, RMND1, RNASEH2A, RNASET2,* ***RNU4ATAC*** *, RNU7-1, ROGDI, RPIA, RSRC1, RUBCN, RUSC2, SACS, SASS6, SCYL1, SCYL2, SELENOI, SEPSECS, SERAC1, SGPL1, SGSH,* ***SIL1*** *, SLC12A1, SLC12A3, SLC17A5, SLC18A2, SLC19A2, SLC19A3, SLC1A4, SLC25A1, SLC25A13, SLC25A15, SLC25A19, SLC25A20, SLC25A3, SLC25A36, SLC25A42, SLC25A46, SLC26A4, SLC35A1, SLC35C1, SLC39A8, SLC44A1, SLC45A1, SLC46A1, SLC4A10, SLC5A6, SLC6A19, SLC6A9, SLC9A1, SMARCAL1, SMC5, SMG9, SMO, SMPD4, SNAP29,* ***SNAPC4*** *, SNORD118, SNX14, SPTBN4, SQOR, SRD5A3, ST3GAL5, STAMBP, STAR, STAT2, STIL, STT3B, STX11, STXBP2, SUCLA2, SUCLG1, SUMF1, SUOX, SURF1, SVBP, TAF13, TAF2, TAF8, TANGO2, TARS2, TASP1, TBC1D20, TBC1D23,* ***TBC1D7*** *, TBCD, TBCE, TBCK, TBX19, TCIRG1, TDP2, TECPR2,* ***TEFM*** *, TELO2,* ***TGDS*** *, THG1L, THOC6, THUMPD1, TIMM50, TIMMDC1, TMCO1, TMEM147, TMEM165, TMEM222, TMEM70, TMEM94, TMTC3, TMX2, TOE1,* ***TOP6BL*** *, TP53RK,* ***TP73*** *, TPK1, TPP1, TRAPPC10, TRAPPC11, TRAPPC12, TRAPPC2L, TRAPPC9,* ***TRIM2*** *, TRIP13, TRIT1, TRMT1, TRMT10A, TRMT5, TRNT1, TRPM6, TSEN15, TSEN2, TSFM, TSPYL1, TTC5, TTI1,* ***TTI2*** *, TUBGCP6, TYROBP,* ***UBE3B*** *, UBE3C, UBE4A, UBR7, UFC1, UFM1, UNC13D, UNC80, UPB1, UQCC2, USP18, USP53, VARS2,* ***VDR*** *, VLDLR,* ***VMA22*** *, VPS13A, VPS13B, VPS13D, VPS35L, VPS41, VPS51, VPS53, WASHC4,* ***WBP4*** *, WDR62, WDR73,* ***WDR81*** *,* ***WDR83OS*** *,* ***WLS*** *, XPNPEP3, YIF1B, YRDC, ZBTB11, ZFYVE26, ZMPSTE24, ZNF142, ZNF335, ZNF526, ZNF668, ZNFX1, ZNHIT3*
AR | SMu	*PAX7*
PR	***DHRSX***
SMu	***MAP3K3***
SMu | XLD	*SLC35A2*
XL	***ABCB7*** *, CNKSR2, DCX, FRMPD4, GLA, GLRA2, HUWE1, NONO, OTC,* ***RLIM*** *, RNF113A, SLITRK2, TFE3, THOC2, XK, ZDHHC9*
XLD	***ACSL4*** *, AMER1, BCOR,* ***CCNQ*** *, CLCN4, GDI1, HCCS, HNRNPH2, HSD17B10, IQSEC2, KDM6A, MSL3, NEXMIF, PDHA1,* ***RPS6KA3*** *, TCEAL1, WDR45, NHS, SLC9A6, FMR1,* ***PLS3***
XLR	*ABCD1, AIFM1, ANOS1, AP1S2,* ***ARSL*** *, ATP6AP1, ATP7A, AVPR2, BCAP31, BCORL1,* ***C1GALT1C1*** *,* ***CD40LG*** *, CHRDL1, CUL4B, DLG3, EIF2S3, FAM50A, FGD1, FTSJ1, GK,* ***GPC3*** *, GRIA3, HCFC1, HPRT1, IDS, IL1RAPL1, KDM5C, KIF4A, KLHL15, LAGE3,* ***LAS1L*** *,* ***MAGT1*** *, MAOA, NDUFA1, OCRL, OGT, OPHN1, OTUD5, PAK3, PGK1, PHF6, PLP1,* ***POLA1*** *, PQBP1, RAB39B, RBM10,* ***SH2D1A*** *, SLC6A8,* ***SLC9A7*** *, SMS, SSR4, STS, SYP, TAF1, UBE2A, USP27X,* ***WNK3*** *,* ***ZRSR2*** *, CASK,* ***PRPS1***
XLD | XLR	*NAA10, IKBKG, ATRX, EBP, MED12, NSDHL, DDX3X, NDP, USP9X, ZC4H2*
UN	***CD2AP*** *, COG8, EMX2, GNAQ, IDH2,* ***IKZF2*** *, KCNK9,* ***NR4A3*** *, PIK3CA, SEZ6,* ***SEZ6L*** *,* ***SEZ6L2*** *, SPR*

AD, autosomal dominant; AR, autosomal recessive; XL, X-linked; XLD, X-linked dominant; XLR, X-linked recessive; DR, digenic recessive; Mu, multifactorial inheritance; SMu, somatic mutation; SMo, somatic mosaicism; DD, digenic dominant; PR, psuedoautosomal recessive; UN, unknown. Genes that have been identified in this study that were not present within the Zhang MW, et al. [1] have been highlighted in bold.

### Predicted epilepsy associated genes

A total of 981 genes were categorised as predicted epilepsy associated genes in this study compared with the study by Zhang MW, et al. [1] who identified 364 neurodevelopment-related genes. These 981 genes were listed according to their mode of inheritance as stated within each genetic database with the majority having multiple modes of inheritance. Common modes of inheritance include autosomal recessive inheritance (20.9%), autosomal dominant inheritance (18.7%), and unknown mode of inheritance (68.2%). Notable phenotypes for epilepsy genes include unknown phenotype (16.6%), mitochondrial dysfunction (4.5%), encephalopathy (3.2%), neurodevelopmental disorder (1.1%) and immunodeficiency (1.1%).

**TABLE 3:** Predicted epilepsy associated genes.

Inheritance	Genes
AD	***ABCC11*** *, AKAP9, ALG10B,* ***APOL2*** *,* ***APOL4*** *,* ***APOLD1*** *, ATP11A, ATP2A2, BAP1, BPTF, CACNA1H, CACNB4, CACNG2, CALM3,* ***CHI3L1*** *, COL4A1, COL4A2,* ***CST3*** *, CTLA4,* ***DAOA*** *,* ***DISC2*** *,* ***DNASE1*** *, DRD3,* ***EDNRA*** *, EFHC1,* ***EIF2AK1*** *,* ***ELP4*** *,* ***EPB41L1*** *, ERMARD, FBP2,* ***FCGR2B*** *, FGFR2,* ***FTH1*** *, GABBR2, GABRD, GAL, GNB2, HCN2, HCN4, HNF1B, HTR2A, IRF3, KCNN2, KDM1A, KIF5A, LRRK2, MAP1B,* ***NEUROD1*** *, NOL3, NOTCH3, NRAS, PAK2,* ***PDGFRB*** *, PHOX2B,* ***PLAU*** *, PRNP, PSEN1, PTEN, RANBP2, RAPGEF2,* ***REST*** *,* ***RET*** *, RORB, RTN4R,* ***SAMD9L*** *, SCN8A, SHANK3, SMARCA4, SMARCB1, SMARCC1, SMARCE1, SNORA31, SPECC1L, SPTSSA, SYN2,* ***TNFRSF1A*** *, TNRC6A, TRPM3, TRRAP, TSC2, TTR,* ***USP25*** *, WDFY3, YEATS2, ZIC1*
AD | AR	*CASR, SLC2A1, TREX1, AARS1, ANTXR1, APOE,* ***ATR*** *, BUB1B, CCDC88C,* ***CILK1*** *, CLCN2,* ***CRELD1*** *, DHTKD1, DMXL2,* ***ESR1*** *,* ***EVC*** *,* ***FCGR2A*** *,* ***HBB*** *, IFT140, IL6ST, KCNQ1,* ***LBR*** *, LMAN2L,* ***MPO*** *, NAGLU,* ***PIEZO2*** *, POLE, PRODH, PSAP, RELN, RYR1,* ***SLC4A1*** *, TBK1,* ***TNFRSF11A*** *, UCHL1, UFSP2, WFS1, SLC12A5,* ***CD46*** *, RNF213, TICAM1, TLR3, CFH,* ***CFHR1*** *,* ***CFHR3*** *,* ***ACKR1*** *, COQ2, CPT2, ATP5F1A, CAMK2A, CDH2, DHDDS,* ***EPO*** *,* ***ERBB3*** *, FIG4, GJC2, GRIA1,* ***HNF1A*** *, HTRA1,* ***IFNG*** *, LMNB2,* ***LRP1*** *, MDM2, MTHFR,* ***PIEZO1*** *, POLG2,* ***PTPN22*** *, RAD21, ROBO1, SCN5A, TGFB1, THAP11,* ***TNF*** *,* ***NEK1*** *, UGT1A1*
AD | AR | Mu	***GBA1*** *, F2, POMC,* ***HLA-DQB1***
AD | AR | DR	***SLC36A2*** *, PIK3CD*
AD | AR | SMu	***SUFU*** *,* ***ELP1*** *, BRCA2, ATM, MINPP1, ERCC6*
AD | AR | SMu | Mu	*IL6*
AD | SMu	*TP53, CBL*
AD | Mu	*NOS3, TBP, MAPT, GABRA2, NOD2*
AR	*ABCA5, ABCB1,* ***ACE*** *,* ***ACER3*** *,* ***ACP2*** *,* ***AHSG*** *, ALG14, AMPD2, AP3D1,* ***AQP4*** *, ARNT2, ATPAF2, B9D2, BCL10, CAMLG,* ***CCT5*** *,* ***CD36*** *, CDC40, CDK5,* ***CDK6*** *, CENPE, CEP290,* ***CEP63*** *,* ***CHP1*** *, CHRNA3, CNP, CNTNAP2, COG2, COQ5, COX8A,* ***CPAP*** *, CPS1, DALRD3,* ***DBR1*** *, DPM3, EGF, EN1, EXOC8,* ***FBXO31*** *, FTO, GET4, GGT1, GUF1, HERC2, HEXA, HMOX1, HTRA2, IBA57, IFNAR2, INTS8, KARS1, KIAA0753, KIF7, LAMA5, LARS1, LSM11,* ***MAL*** *, MCM8, MDH1, MRPL12, MTHFD1, MTR,* ***MTRR*** *, MYO1H, NARS2, NAT8L, NDUFA13,* ***NDUFA4*** *, NIN, NRXN1, NUP133, NUP214, PAH, PCSK1, PDCD6IP, PEX3,* ***PHYKPL*** *, PIGG, PNPLA8, POMK, PPA2, PRDM8, PRIMA1,* ***PSMB1*** *,* ***RFX5*** *,* ***RNH1*** *, RNU12,* ***RRP7A*** *, SARDH, SEC31A,* ***SEL1L*** *, SHQ1,* ***SIK3*** *,* ***SLC19A1*** *, SLC24A4,* ***SLC24A5*** *, SLC25A10, SLC28A1, SOBP, SYT14, TDP1, TMEM67,* ***TREM2*** *,* ***TSEN34*** *, TSEN54,* ***TSPEAR*** *,* ***TSPOAP1*** *, TXN2,* ***UQCC3*** *, VPS11, WIPI2,* ***ZPR1***
AR | Mu	***HLA-DQA1***
DR	*KNSTRN*
XL	***G6PD*** *, NLGN3*
XLD	*NDUFB11*
XLR	*ATP6AP2, HS6ST2, L1CAM, MBTPS2, MID2, PTCHD1,* ***RBMX*** *, RPL10, TMLHE*
XLD | XLR	*DKC1, FLNA, MECP2, OFD1*
UN	***ABCC1*** *, ABCC12, ABCC2,* ***ABCC5*** *,* ***ABCG2*** *,* ***ACHE*** *, ACMSD,* ***ACOD1*** *, ACOT7, ADA,* ***ADAM23*** *,* ***ADCY9*** *,* ***ADGRB3*** *,* ***ADGRL3*** *,* ***ADIPOQ*** *,* ***ADORA1*** *, ADORA2A, ADRA2B,* ***AGAP2*** *,* ***AGER*** *,* ***AGMO*** *,* ***AGTR1*** *, AGTR2,* ***AHR*** *,* ***AJAP1*** *,* ***AK5*** *, AKAP5,* ***ALB*** *,* ***ALDH2*** *,* ***AMPH*** *,* ***ANKK1*** *, ANO3,* ***ANO4*** *,* ***ANXA7*** *,* ***APLN*** *,* ***APTX*** *,* ***AQP1*** *,* ***AQP9*** *,* ***ARF6*** *,* ***ARHGAP44*** *, ARHGEF15,* ***ARHGEF5*** *,* ***ARL6IP6*** *,* ***ARPC2*** *,* ***ASIC2*** *,* ***ASIC3*** *,* ***ASNSD1*** *,* ***ATF2*** *,* ***ATF3*** *,* ***ATF5*** *,* ***ATG5*** *,* ***ATL1*** *,* ***ATOX1*** *,* ***ATP10A*** *,* ***ATP2B2*** *,* ***AVP*** *,* ***AZGP1*** *,* ***BACE1*** *,* ***BAK1*** *,* ***BCHE*** *, BCL11A,* ***BCL2A1*** *, BDNF,* ***BMAL1*** *,* ***BORCS5*** *, BRD2, BRWD3, BSN,* ***C1orf94*** *, C3, C9orf72,* ***CACNG4*** *,* ***CALB1*** *,* ***CALB2*** *,* ***CALHM1*** *,* ***CAMK4*** *, CAPN1,* ***CAPN2*** *, CAPZA2,* ***CASP1*** *,* ***CASP3*** *,* ***CAT*** *,* ***CBLN1*** *,* ***CBX1*** *, CCDC186,* ***CCDC82*** *,* ***CCL2*** *,* ***CCL4*** *, CCN3,* ***CCR5*** *,* ***CCS*** *,* ***CCT4*** *,* ***CCT6A*** *,* ***CCT7*** *,* ***CCT8*** *,* ***CD34*** *,* ***CD40*** *,* ***CD44*** *,* ***CDIP1*** *,* ***CDK5R1*** *,* ***CDK5RAP3*** *,* ***CDKN2B-AS1*** *,* ***CDYL*** *, CELF4, CELSR1, CELSR3,* ***CFL1*** *, CHD4, CHL1, CHRFAM7A, CHRM1,* ***CHRM3*** *,* ***CHRNA5*** *, CHRNA7,* ***CISH*** *, CLCN7,* ***CLDN5*** *,* ***CLDND1*** *,* ***CLEC16A*** *, CLIP1, CLOCK,* ***CLPTM1*** *, CLTCL1,* ***CLU*** *, CNOT2,* ***CNOT9*** *,* ***CNR1*** *,* ***CNRIP1*** *,* ***COL1A1*** *, COL6A2,* ***COPZ2*** *,* ***COX20*** *, CP,* ***CPEB4*** *,* ***CPLX2*** *,* ***CR1*** *,* ***CREB1*** *,* ***CREM*** *, CRH,* ***CRMP1*** *,* ***CRP*** *, CSMD1, CSMD3, CSNK1E, CSNK1G1,* ***CSNK1G2*** *, CTNND2,* ***CXCL13*** *,* ***CXCL8*** *,* ***CXCR2*** *,* ***CXCR5*** *, CYFIP1,* ***CYP19A1*** *,* ***CYP1A1*** *,* ***CYP1A2*** *,* ***CYP24A1*** *,* ***CYP2C19*** *,* ***CYP2D6*** *,* ***CYP3A4*** *,* ***CYP3A5*** *,* ***DAPK1*** *,* ***DBN1*** *,* ***DBP*** *, DCAF13, DCLK2,* ***DDX54*** *, DENND2B,* ***DFFB*** *, DGKD, DHRS9,* ***DIPK2A*** *,* ***DIRAS1*** *,* ***DISC1*** *, DLG2, DLGAP2,* ***DLGAP3*** *,* ***DLX1*** *, DMD,* ***DNAJA3*** *,* ***DOC2A*** *,* ***DPYSL2*** *,* ***DRD1*** *, DSCAM,* ***DTNBP1*** *,* ***DUSP4*** *,* ***EDN1*** *,* ***EFHC2*** *,* ***EGFR*** *,* ***EGR1*** *,* ***EIF2S1*** *, ELFN1,* ***ENO2*** *,* ***EPHX1*** *,* ***EPM2AIP1*** *, ERBB4,* ***ERMN*** *,* ***ERN1*** *,* ***ESM1*** *,* ***EZR*** *,* ***FAAH*** *, FAAH2,* ***FAM3C*** *,* ***FANCL*** *, FAT1,* ***FGA*** *, FGB,* ***FGF2*** *,* ***FGF22*** *,* ***FGF7*** *,* ***FGG*** *,* ***FKBP5*** *,* ***FLT1*** *, FN1,* ***FNDC5*** *,* ***FOS*** *,* ***FOXO3*** *,* ***FOXO4*** *, FURIN,* ***FYN*** *, FZD9,* ***GABARAP*** *, GABRA4, GABRA6, GABRE,* ***GABRR1*** *, GABRR2,* ***GAD2*** *,* ***GAPDH*** *,* ***GAS1*** *,* ***GC*** *,* ***GDNF*** *,* ***GEMIN5*** *,* ***GHRH*** *, GHRL,* ***GJB1*** *, GJD2,* ***GLO1*** *, GLP1R,* ***GNB3*** *,* ***GNG3*** *,* ***GOLIM4*** *,* ***GPC4*** *, GPR37L1,* ***GPX1*** *, GRIK1,* ***GRIK5*** *, GRINA,* ***GRM2*** *, GRM3, GRM4,* ***GRM5*** *,* ***GRPR*** *,* ***GSK3A*** *,* ***GSK3B*** *, GSN,* ***GSTA1*** *,* ***GSTA4*** *, GSTM1,* ***GSTP1*** *,* ***GSTT1*** *,* ***GYG2*** *,* ***GYPA*** *,* ***GYPB*** *,* ***GYPC*** *,* ***HCN3*** *,* ***HCRT*** *,* ***HDAC2*** *,* ***HIF1A*** *, HIP1,* ***HLA-B*** *,* ***HLA-DRB1*** *,* ***HLF*** *,* ***HMGB1*** *,* ***HOMER1*** *,* ***HP*** *,* ***HRH3*** *,* ***HSPA8*** *,* ***HSPB1*** *,* ***HSPBAP1*** *, HSPE1, HTR1A,* ***HTR1B*** *, HTR2C,* ***HTR3A*** *, HTR6,* ***HTR7*** *,* ***HULC*** *,* ***ICAM1*** *,* ***ICAM5*** *, IDH1,* ***IDO1*** *,* ***IFI44L*** *,* ***IFNA1*** *,* ***IGF1*** *,* ***IGF1R*** *, IL10,* ***IL17A*** *,* ***IL17RA*** *,* ***IL18*** *,* ***IL1A*** *, IL1B,* ***IL1R1*** *,* ***IL1RN*** *,* ***IL2*** *,* ***IL33*** *,* ***IL4*** *,* ***IL7R*** *,* ***IMMT*** *,* ***IMPA2*** *, INPP4A,* ***IRAK1*** *,* ***IRS1*** *,* ***IRS2*** *,* ***ITGA2*** *,* ***ITSN1*** *, JMJD1C, JRK,* ***JUN*** *,* ***KCNA3*** *,* ***KCNA4*** *,* ***KCNA6*** *, KCNAB1, KCNAB2,* ***KCNAB3*** *, KCND2,* ***KCNH3*** *,* ***KCNH7*** *,* ***KCNIP3*** *,* ***KCNJ3*** *,* ***KCNJ8*** *,* ***KCNK18*** *,* ***KCNK2*** *,* ***KCNK3*** *, KCNMB1,* ***KCNMB4*** *,* ***KCNN4*** *,* ***KCNV1*** *, KCNV2,* ***KDM2B*** *,* ***KEAP1*** *,* ***KIF16B*** *,* ***KL*** *, KLHL20,* ***KLRC1*** *,* ***KLRK1*** *,* ***LEP*** *, LEPR,* ***LGI2*** *,* ***LGMN*** *,* ***LINC01621*** *,* ***LOC101927235*** *,* ***LOC102724058*** *,* ***LOC108228197*** *,* ***LOC108228198*** *,* ***LOC108228208*** *,* ***LOC108228209*** *,* ***MAOB*** *, MAP2, MAPK10,* ***MARK2*** *, MAST4,* ***MBP*** *, MC3R, ME2,* ***MED1*** *,* ***MGMT*** *, MICAL1,* ***MICU2*** *,* ***MIR124-1*** *, MIR128-1,* ***MIR128-2*** *,* ***MIR129-1*** *,* ***MIR129-2*** *,* ***MIR132*** *,* ***MIR134*** *,* ***MIR135B*** *,* ***MIR139*** *,* ***MIR145*** *,* ***MIR146A*** *,* ***MIR148A*** *,* ***MIR153-1*** *,* ***MIR153-2*** *,* ***MIR155*** *,* ***MIR15A*** *,* ***MIR181A1*** *,* ***MIR196B*** *,* ***MIR199A1*** *,* ***MIR199A2*** *,* ***MIR204*** *,* ***MIR211*** *,* ***MIR212*** *,* ***MIR217*** *,* ***MIR218-1*** *,* ***MIR219A1*** *,* ***MIR29A*** *,* ***MIR335*** *,* ***MIR372*** *, MLLT3,* ***MMP2*** *,* ***MMP3*** *,* ***MMP8*** *,* ***MMP9*** *,* ***MT-ATP6*** *,* ***MT-ATP8*** *,* ***MT-CO2*** *,* ***MT-ND1*** *,* ***MT-ND4*** *,* ***MT-ND5*** *,* ***MT-ND6*** *,* ***MT-RNR1*** *,* ***MT-RNR2*** *,* ***MT-TF*** *,* ***MT-TL1*** *,* ***MT-TS1*** *,* ***MT2A*** *, MUSK,* ***MVP*** *,* ***MYH10*** *,* ***NAPA*** *,* ***NAT2*** *,* ***NAV1*** *,* ***NCAM1*** *, NCOR2,* ***NEAT1*** *,* ***NES*** *,* ***NFE2L1*** *,* ***NFIX*** *,* ***NFKB1*** *, NIPA2,* ***NKAIN1*** *,* ***NKAIN2*** *,* ***NKAIN4*** *, NLGN1,* ***NLRP1*** *,* ***NNMT*** *,* ***NOS1*** *,* ***NOS2*** *,* ***NOTCH4*** *,* ***NPAP1*** *,* ***NPPB*** *,* ***NPPC*** *,* ***NPY*** *,* ***NPY1R*** *,* ***NPY2R*** *,* ***NR1I2*** *,* ***NR1I3*** *,* ***NR3C1*** *,* ***NRDC*** *,* ***NREP*** *,* ***NRG1*** *, NRXN2, NTF3, NTNG1,* ***NUCB2*** *,* ***NUSAP1*** *,* ***NWD1*** *, OGDHL,* ***OLIG2*** *,* ***OMA1*** *,* ***OPALIN*** *, OPRM1,* ***OPTN*** *,* ***OSBPL11*** *,* ***OTX1*** *,* ***OXT*** *,* ***P2RX3*** *,* ***P2RX7*** *,* ***P2RY1*** *,* ***P2RY12*** *,* ***P2RY2*** *,* ***PADI4*** *,* ***PADI6*** *,* ***PAQR8*** *,* ***PARP6*** *, PAX6, PCDH7,* ***PCMT1*** *,* ***PER2*** *,* ***PEX5L*** *,* ***PGF*** *,* ***PHF24*** *,* ***PHLDA1*** *,* ***PHOX2A*** *,* ***PIK3C2B*** *, PIK3R4,* ***PIN1*** *,* ***PJA1*** *, PLA2G4A,* ***PLAT*** *,* ***PLAUR*** *, PLCB4,* ***PLCG1*** *,* ***PNOC*** *,* ***POU2AF1*** *,* ***PPARG*** *,* ***PPFIA1*** *,* ***PPP2R2C*** *, PPP5C,* ***PPT2*** *, PRICKLE2, PRKD1, PRL, PROM1,* ***PRR15L*** *, PTGS2, PTPRD,* ***PVALB*** *,* ***PWP2*** *,* ***QKI*** *,* ***RAB40AL*** *,* ***RACK1*** *,* ***RALBP1*** *,* ***RAP1A*** *,* ***RARRES2*** *,* ***RASGRF1*** *, RBFOX1, RBFOX3, RFX3, RFX4,* ***RHOA*** *,* ***RINT1*** *,* ***RNU2-2*** *,* ***RNU5B-1*** *,* ***RPS6KA2*** *,* ***RTN4*** *,* ***S100B*** *,* ***SAA1*** *, SCAMP5,* ***SCN1A-AS1*** *,* ***SCN3B*** *,* ***SEMA7A*** *,* ***SERINC1*** *,* ***SERINC2*** *,* ***SERINC5*** *,* ***SERPINE1*** *,* ***SGK1*** *,* ***SH3BGRL2*** *,* ***SH3GL2*** *, SHANK2,* ***SHH*** *, SHROOM4,* ***SIRT1*** *,* ***SKAP1*** *,* ***SLC13A1*** *, SLC16A4, SLC16A7, SLC1A1, SLC22A1, SLC29A1,* ***SLC39A7*** *,* ***SLC4A5*** *,* ***SLC5A11*** *, SLC6A3, SLC6A4,* ***SLC7A10*** *, SLC7A11, SLC9A9,* ***SLIT2*** *,* ***SLITRK3*** *,* ***SLITRK5*** *,* ***SMAD3*** *, SMG6,* ***SNHG1*** *,* ***SNN*** *,* ***SNRPD1*** *,* ***SNX11*** *,* ***SNX25*** *,* ***SOCS1*** *,* ***SOD1*** *,* ***SOD2*** *,* ***SORBS2*** *,* ***SORCS2*** *, SORCS3, SOX11,* ***SOX7*** *,* ***SP1*** *, SRGAP2, SRPX2,* ***SRR*** *,* ***SSTR2*** *,* ***ST8SIA1*** *,* ***STAT3*** *,* ***STIM1*** *,* ***STIM2*** *,* ***STK24*** *,* ***STMN1*** *,* ***STX1A*** *, STXBP5L, STYXL1, SUCO,* ***SV2B*** *,* ***SYNRG*** *,* ***SYT11*** *,* ***SYVN1*** *,* ***TAC1*** *,* ***TACR1*** *, TAP1,* ***TBC1D32*** *,* ***TEF*** *,* ***TERT*** *,* ***TET1*** *,* ***TET2*** *, TFAM,* ***TGFBR2*** *,* ***THBS1*** *,* ***THBS2*** *,* ***TIMP1*** *,* ***TIMP2*** *,* ***TIMP4*** *,* ***TIRAP*** *,* ***TLE1*** *,* ***TLN2*** *,* ***TLR4*** *,* ***TLR7*** *,* ***TMEFF1*** *,* ***TMEM25*** *, TMEM63B,* ***TNFRSF13C*** *,* ***TNFRSF1B*** *,* ***TNFSF13B*** *, TNK2,* ***TOMM40*** *,* ***TOP2A*** *, TOP3B,* ***TPH2*** *,* ***TRIM3*** *, TRMT44,* ***TRPC3*** *,* ***TRPC4*** *, TRPC6,* ***TRPM2*** *,* ***TRPM7*** *,* ***TRPV1*** *,* ***TRPV4*** *,* ***TSPO*** *,* ***TUBG2*** *,* ***TUG1*** *, TXNRD1,* ***U2AF1*** *, UBA1,* ***UBC*** *,* ***UBQLN1*** *, UBR4, UBR5,* ***UCP2*** *,* ***UGT1A4*** *,* ***UGT1A6*** *, UGT1A9,* ***UGT2B7*** *, UNC13B, UPF1,* ***USF1*** *,* ***VEGFA*** *,* ***VEZT*** *, VRK2,* ***VWF*** *,* ***WHCR*** *,* ***WNT8B*** *, YWHAE,* ***YWHAQ*** *,* ***YWHAZ*** *, ZDHHC15,* ***ZFAS1*** *, ZFHX3,* ***ZFYVE27*** *,* ***ZNF143*** *,* ***ZNF385D*** *,* ***ZSCAN25*** *, COMT*

AD, autosomal dominant; AR, autosomal recessive; XL, X-linked; XLD, X-linked dominant; XLR, X-linked recessive; DR, digenic recessive; Mu, multifactorial inheritance; SMu, somatic mutation; SMo, somatic mosaicism; DD, digenic dominant; PR, psuedoautosomal recessive; UN, unknown. Genes underlined did not have a phenotype registered within any database. Genes that have been identified in this study that were not present within the Zhang MW, et al. [1] have been highlighted in bold.

### Frequently reported/identified epilepsy genes

A total of 86 epilepsy genes have been reported/identified in all three databases analysed in this study and have been classified as epilepsy genes. Along with each associated phenotypes and mode of inheritance. Figure 1A states that there are 90 genes that have been identified and filtered from all three databases. The genes ABAT and DPM2 were excluded due to their placement in the category epilepsy associated genes and the genes GABRD and SLC12A5 were excluded due to their placement in the category predicted epilepsy associated genes.

Notable phenotypes for the common epilepsy genes (Table 4) include encephalopathy (48.8%), neurodevelopmental disorder (12.8%), myoclonic (10.5%), microcephaly (8.1%) and hypotonia (3.5%). When comparing the phenotype distribution of the frequently reported/identified epilepsy genes (Table 4) to the phenotype distribution of epilepsy genes (Table 1) there is some variation. There is a 5.1% increase with the encephalopathy phenotype compared to the epilepsy genes found in Table 1. There is a 10.7% decrease with the neurodevelopmental disorder phenotype, a 2.5% increase with the progressive myoclonic phenotype, a 0.3% decrease with the microcephaly phenotype and a 3.6% decrease with the hypotonia phenotype compared to the epilepsy genes phenotype distribution.

**TABLE 4:** Epilepsy genes found in all databases.

Gene	Inheritance	Phenotype/s
*ALDH7A1*	AR	• Epilepsy, early-onset, vitamin B6-dependent
*ALG13*	XL	• Developmental and epileptic encephalopathy
*ARX*	XLR | XL	• Developmental and epileptic encephalopathy • Hydranencephaly with abnormal genitalia • Intellectual developmental disorder • Lissencephaly • Partington syndrome • Proud syndrome
*ATP1A2*	AD | AR	• Alternating hemiplegia of childhood • Developmental and epileptic encephalopathy • Fetal akinesia, respiratory insufficiency, microcephaly, polymicrogyria, and dysmorphic facies
*BRAT1*	AR	• Neurodevelopmental disorder with cerebellar atrophy and with or without seizures • Rigidity and multifocal seizure syndrome, lethal neonatal
*CACNA1E*	AD	• Developmental and epileptic encephalopathy
*CAD*	AR	• Developmental and epileptic encephalopathy
*CERS1*	AR	• Epilepsy, progressive myoclonic
*CHRNA2*	AD	• Epilepsy, nocturnal frontal lobe
*CHRNA4*	AD	• Epilepsy, nocturnal frontal lobe
*CHRNB2*	AD	• Epilepsy, nocturnal frontal lobe
*CNNM2*	AD | AR	• Hypomagnesemia, renal • Hypomagnesemia, seizures, and impaired intellectual development
*CSTB*	AR	• Epilepsy, progressive myoclonic
*CUX2*	AD	• Developmental and epileptic encephalopathy
*DEPDC5*	AR | AD	• Developmental and epileptic encephalopathy • Epilepsy, familial focal
*DIAPH1*	AD | AR	• Deafness, with or without thrombocytopenia • Seizures, cortical blindness, microcephaly syndrome
*DLL1*	AD	• Neurodevelopmental disorder with nonspecific brain abnormalities and with or without seizures
*DNM1*	AD | AR	• Developmental and epileptic encephalopathy
*DOCK7*	AR	• Developmental and epileptic encephalopathy
*FGF12*	AD	• Developmental and epileptic encephalopathy
*FRRS1L*	AR	• Developmental and epileptic encephalopathy
*GABRA1*	AD	• Developmental and epileptic encephalopathy
*GABRB1*	AD	• Developmental and epileptic encephalopathy
*GABRB3*	AD	• Developmental and epileptic encephalopathy
*GABRG2*	AD	• Developmental and epileptic encephalopathy • Febrile seizures • Generalized epilepsy with febrile seizures
*GAD1*	AR	• Developmental and epileptic encephalopathy
*GLUL*	AD | AR	• Glutamine deficiency
*GNAO1*	AD	• Developmental and epileptic encephalopathy • Neurodevelopmental disorder with involuntary movements
*GOSR2*	AR	• Epilepsy, progressive myoclonic • Muscular dystrophy, congenital, with or without seizures
*GRIA4*	AD	• Neurodevelopmental disorder with or without seizures and gait abnormalities
*HCN1*	AD	• Developmental and epileptic encephalopathy • Generalized epilepsy with febrile seizures
*ITPA*	AR	• Inosine triphosphatase deficiency • Developmental and epileptic encephalopathy
*KCNA2*	AD	• Developmental and epileptic encephalopathy
*KCNC1*	AD	• Epilepsy, progressive myoclonic
*KCNC2*	AD	• Developmental and epileptic encephalopathy
*KCNH5*	AD	• Developmental and epileptic encephalopathy
*KCNK4*	AD	• Facial dysmorphism, hypertrichosis, epilepsy, intellectual/developmental delay, and gingival overgrowth syndrome
*KCNMA1*	AD | AR	• Epilepsy, idiopathic generalized • Cerebellar atrophy, developmental delay, and seizures • Liang-Wang syndrome • Paroxysmal nonkinesigenic dyskinesia, with or without generalized epilepsy
*KCNQ2*	AD	• Developmental and epileptic encephalopathy • Myokymia • Seizures, benign neonatal
*KCNQ3*	AD | AR	• Seizures, benign neonatal
*KCNT1*	AD	• Developmental and epileptic encephalopathy • Epilepsy nocturnal frontal lobe
*KCTD7*	AR	• Epilepsy, progressive myoclonic, with or without intracellular inclusions
*LGI1*	AD	• Epilepsy, familial temporal lobe
*NACC1*	AD	• Neurodevelopmental disorder with epilepsy, cataracts, feeding difficulties, and delayed brain myelination
*NARS1*	AR | AD	• Neurodevelopmental disorder with microcephaly, impaired language, and gait abnormalities • Neurodevelopmental disorder with microcephaly, impaired language, epilepsy, and gait abnormalities
*NECAP1*	AR	• Developmental and epileptic encephalopathy
*NPRL2*	AD	• Epilepsy, familial focal
*NPRL3*	AD	• Epilepsy, familial focal
*NRROS*	AR	• Seizures, early-onset, with neurodegeneration and brain calcification
*NSRP1*	AR	• Neurodevelopmental disorder with spasticity, seizures, and brain abnormalities
*NUS1*	AR | AD	• Congenital disorder of glycosylation, type 1aa • Intellectual developmental disorder, autosomal dominant 55, with seizures
*PACS2*	AD	• Developmental and epileptic encephalopathy
*PIGB*	AR	• Developmental and epileptic encephalopathy
*PIGF*	AR	• Onychodystrophy, osteodystrophy, impaired intellectual development, and seizures syndrome
*PIGN*	AR	• Multiple congenital anomalies-hypotonia-seizures syndrome
*PIGP*	AR	• Developmental and epileptic encephalopathy
*PIGQ*	AR	• Multiple congenital anomalies-hypotonia-seizures syndrome
*PIGT*	AD | SMu | AR	• Paroxysmal nocturnal hemoglobinuria • Multiple congenital anomalies-hypotonia-seizures syndrome
*PLCB1*	AR	• Developmental and epileptic encephalopathy
*PNKP*	AR	• Charcot-Marie-Tooth disease, type 2B2 • Ataxia-oculomotor apraxia • Microcephaly, seizures, and developmental delay
*PNPO*	AR	• Pyridoxamine 5′-phosphate oxidase deficiency
*PRICKLE1*	AR	• Epilepsy, progressive myoclonic
*PRRT2*	AD	• Convulsions, familial infantile, with paroxysmal choreoathetosis • Episodic kinesigenic dyskinesia • Seizures, benign familial infantile
*QARS1*	AR	• Microcephaly, progressive, seizures, and cerebral and cerebellar atrophy
*SARS1*	AR	• Neurodevelopmental disorder with microcephaly, ataxia, and seizures
*SCARB2*	AR	• Epilepsy, progressive myoclonic, with or without renal failure
*SCN1A*	AD	• Developmental and epileptic encephalopathy • Dravet syndrome • Febrile seizures • Generalized epilepsy with febrile seizures plus • Migraine, familial hemiplegic
*SCN1B*	AD | AR	• Atrial fibrillation • Brugada syndrome • Cardiac conduction defect, nonspecific • Developmental and epileptic encephalopathy • Generalized epilepsy with febrile seizures plus
*SCN3A*	AD	• Developmental and epileptic encephalopathy • Epilepsy, familial focal
*SEMA6B*	AD	• Epilepsy, progressive myoclonic
*SERPINI1*	AD	• Encephalopathy, with neuroserpin inclusion bodies
*SIK1*	AD	• Developmental and epileptic encephalopathy
*SLC1A2*	AD	• Developmental and epileptic encephalopathy
*SLC25A22*	AR	• Developmental and epileptic encephalopathy
*SNAP25*	AD	• Developmental and epileptic encephalopathy
*SPTAN1*	AD	• Developmental and epileptic encephalopathy • Developmental delay with or without epilepsy • Neuronopathy, distal hereditary motor • Spastic paraplegia, with or without cerebellar ataxia
*STX1B*	AD	• Generalized epilepsy with febrile seizures plus
*STXBP1*	AD | AR	• Developmental and epileptic encephalopathy
*SYNJ1*	AR	• Developmental and epileptic encephalopathy • Parkinson disease, early-onset
*SZT2*	AR	• Developmental and epileptic encephalopathy
*TANC2*	AD	• Intellectual developmental disorder with autistic features and language delay, with or without seizures
*TRAPPC4*	AR	• Neurodevelopmental disorder with epilepsy, spasticity, and brain atrophy
*TRAPPC6B*	AR	• Neurodevelopmental disorder with microcephaly, epilepsy, and brain atrophy
*UBA5*	AR	• Spinocerebellar ataxia • Developmental and epileptic encephalopathy
*VARS1*	AR	• Neurodevelopmental disorder with microcephaly, seizures, and cortical atrophy
*WWOX*	AR	• Developmental and epileptic encephalopathy • Esophageal squamous cell carcinoma • Spinocerebellar ataxia

AD, autosomal dominant; AR, autosomal recessive; XL, X-linked; XLD, X-linked dominant; XLR, X-linked recessive; DR, digenic recessive; Mu, multifactorial inheritance; SMu, somatic mutation; SMo, somatic mosaicism; DD, digenic dominant; PR, psuedoautosomal recessive; UN, unknown.

## Discussion

The aim of the present study was to provide a list of the current genes that are or have a predicted association with epilepsy. Previously, a study by Zhang MW, et al. [1] identified 1440 epilepsy associated genes based on OMIM, PubMed and the human genome mutation database (HGMD). The epilepsy keywords that were searched within each database remain the same between this study and the one performed by Zhang MW, et al. The current study identified a list of 2,536 epilepsy associated genes based on experimental and clinical evidence as provided by the three databases: OMIM, ClinGen and PubMed. This was done by evaluating gene-disease associations that involved multiple aspects of evidence and characteristics. The wide acceptance of genes from these three databases allowed for the incorporation of more potentially disease-associated genes, giving researchers a broader catalogue of genes to study. To aid in this endeavour, genes were categorised into three categories based on the level of currently known association to epilepsy.

This study identified 238 genes (Table 1) that are classified as epilepsy genes, which is more than that of Zhang MW, et al. [1] who classified 168 genes as epilepsy genes. It is important to note that the comparison of results made between this study and the study by Zhang MW, et al. [1] used different parameters and databases. Most of the epilepsy genes identified in this study had a phenotype of encephalopathy (43.7%) which is to be expected when looking at a list of genes with the strongest link to epilepsy.

This study identified 1317 genes (Table 2) that are classified as epilepsy associated genes, which is more than that of Zhang MW, et al. [1] who classified 364 neurodevelopment-associated epilepsy genes. Most of these genes had a phenotype of mitochondrial dysfunction (8.9%) and neurodevelopmental dysfunction (6.8%). This category produced the largest list of genes which also produced the largest variation in phenotypes. This explains the lower distribution percentages of each phenotype listed.

This study identified 981 genes (Table 3) that are classified as predicted epilepsy associated genes, which is more than that of Zhang MW, et al. [1] who classified 974 genes as epilepsy-related genes. Most of these genes had an unknown phenotype (16.6%) but were still classified into potential epilepsy associated genes by the databases looked at in this study.

Finally, we provided a list of 86 genes (Table 4) that are categorised as epilepsy genes and were identified in all databases. Summarised phenotypes have been included for each of these genes. Some of the least published genes from this list include *FRRS1L, NECAP1, NSRP1, QARS1* and *VARS1*. The ferric chelate reductase 1 like protein (*FRRS1L*) is a protein coding gene that encodes an important receptor protein in the brain and modulates glutamate signalling. Loss of the *FRRS1L* gene has shown to disrupt synaptic AMPA receptor function resulting in neurodevelopmental, motor and cognitive abnormalities [5]. Variants in this gene have caused epileptic encephalopathy with hyperkinetic movements [6] and epileptic-dyskinetic encephalopathy [7]. NECAP endocyctosis associated 1 (*NECAP1*) is a coding gene that produces a protein that localises to clathrin-coated vesicles, where it binds components of the adapter protein complexes and aids in endocytosis. This gene has been linked to early onset epileptic encephalopathy through next-generation sequencing [8]. However, the genetic aetiology of *NECAP1* remains to be fully understood. *NSRP1* is involved in developmental process and regulation of alternative mRNA splicing, via spliceosome. This gene has been associated with breast cancer [9], spastic cerebral palsy and epilepsy [10]. These papers are both recent and introductory into the investigation of connecting this gene to an association with a disease phenotype. *QARS1* is involved with dendritic and axonal development. This gene has been associated with epileptic encephalopathy [11, 12] and breast cancer [13]. Finally, *VARS1* shares similar functions with *QARS1* in which they both encode tRNA synthetases but effect different tRNA functions. *VARS1* has been associated with improving melanoma immunotherapy efficacy [14], hepatocellular carcinoma [15] and neurodevelopment disorder [16].

This study has several limitations that should be taken into consideration. To generate this list, all genes were included from all three databases whether they showed a direct link to epilepsy or not. Such genes, for example, that did not have an established phenotype were grouped into the predicted epilepsy associated genes category. These genes have been underlined within Table 3 and makeup approximately 16% of the genes in the Predicted associated epilepsy gene category. Comparisons were made throughout the paper with a previous paper by Zheng MW, et al. [1] and it should be noted that our paper used different parameters and databases to this paper. This comparison is to show a general trend of how our understanding of epilepsy associated genes are progressing. Finally, it should be noted that there may be potential biases in such data produced from all three databases.

In conclusion, the present study updates the list of epilepsy associated genes based on currently available genetic databases, to provide a catalogue of genetic information in the hopes to better understand the aetiology of epilepsy and possibly other neurodevelopmental diseases. This list showcases a range of inheritance modes and phenotypes of the most common genes to aid in this endeavour.

## Footnotes

1
https://www.omim.org/

2
https://clinicalgenome.org/

3
https://pubmed.ncbi.nlm.nih.gov/
