# Supplementary material for: Genetics of epilepsy
Source: Exp Biol Med (Maywood). 2026 Mar 19;251:10933. doi: 10.3389/ebm.2026.10933 (PMC13075117; doi:10.3389/ebm.2026.10933)
Supplement: Supplementary file 1 [file Table1.docx]

**Supplementary material**

Table 1. Criteria for a gene to be placed into each gene category based on the database.

| **Category** | **OMIM** | **ClinGen** | **PubMed** |
| --- | --- | --- | --- |
| Epilepsy genes | - Phenotype map key = 3   And   - Phenotype contains epilepsy key word/s | - Gene is labelled as Definitive | - Phenotype contains epilepsy key word/s   And   - Phenotype contains strong-evidence word/s |
| Epilepsy associated genes | - Phenotype map key = 1,3 and/or 4   And   - Phenotype lacks epilepsy key word/s | - Gene is labelled as Moderate | - Phenotype contains epilepsy key word/s   And   - Phenotype lacks strong-evidence word/s |
| Predicted epilepsy associated genes | - Phenotype is blank   Or   - Phenotype starts with ?,{, [, / | - Gene is labelled as Limited | - Phenotype lacks epilepsy key word/s |

Phenotype map keys include: Key 1, phenotype mapped to a locus, gene unknown; Key 2, confirmed mendelian phenotype, molecular basis unknown; Key 3, molecular basis known (confirmed pathogenic variants); Key 4, statistical association only, gene-phenotype link not confirmed. Epilepsy key words include: epilepsy, epilepsies, epileptic, seizure, seizures, convulsion, convulsions. OMIM prefix symbols include: ?, uncertain gene-phenotype relationship; {}, susceptibility phenotypes; [], phenotypes mapped by linkage only; /, ambiguous or alternative phenotypes designations.
